# Hydrogel Microparticles for Fluorescence Detection of miRNA in Mix-Read Bioassay

**DOI:** 10.3390/s21227671

**Published:** 2021-11-18

**Authors:** Alessia Mazzarotta, Tania Mariastella Caputo, Edmondo Battista, Paolo Antonio Netti, Filippo Causa

**Affiliations:** 1 Center for Advanced Biomaterials for Healthcare, Istituto Italiano di Tecnologia, L.go Barsanti e Matteucci, 53, 80125 Naples, Italy; alessia.mazzarotta@gmail.com (A.M.); taniacaputo87@gmail.com (T.M.C.); nettipa@unina.it (P.A.N.); causa@unina.it (F.C.); 2 Interdisciplinary Research Centre on Biomaterials (CRIB), Università degli Studi di Napoli “Federico II”, P.le Tecchio 80, 80125 Naples, Italy; 3 Dipartimento di Ingegneria Chimica del Materiali e della Produzione Industriale, Università degli Studi di Napoli “Federico II”, P.le Tecchio 80, 80125 Naples, Italy

**Keywords:** hydrogel microparticles, 3D recognition, miRNA detection, mix-read bioassay

## Abstract

Herein we describe the development of a mix-read bioassay based on a three-dimensional (3D) poly ethylene glycol—(PEG)-hydrogel microparticles for the detection of oligonucleotides in complex media. The key steps of hydrogels synthesis and molecular recognition in a 3D polymer network are elucidated. The design of the DNA probes and their density in polymer network were opportunely optimized. Furthermore, the diffusion into the polymer was tuned adjusting the polymer concentration and consequently the characteristic mesh size. Upon parameters optimization, 3D-PEG-hydrogels were synthetized in a microfluidic system and provided with fluorescent probe. Target detection occurred by double strand displacement assay associated to fluorescence depletion within the hydrogel microparticle. Proposed 3D-PEG-hydrogel microparticles were designed for miR-143-3p detection. Results showed 3D-hydrogel microparticles with working range comprise between 10^−6^–10^−12^ M, had limit of detection of 30 pM and good specificity. Moreover, due to the anti-fouling properties of PEG-hydrogel, the target detection occurred in human serum with performance comparable to that in buffer. Due to the approach versatility, such design could be easily adapted to other short oligonucleotides detection.

## 1. Introduction

Many analytical methods have been developed so far with the aim to identify oligonucleotide biomarkers in human fluids [1]. However, the majority require repeated separation and washing steps which is time-consuming, expensive, and subject to errors. For these reasons, in the last year, many no-wash or mix-read biosensors have been developed [1,2] and have led the way toward innovative and sensitive devices for Point of Care (POC) applications. Mix-read (or mix & read) bioassays are easily made by just mixing the sample solution with a signal generator probe (biosensor) and reading the detectable signal in solution or on solid surface. The signal is generated following the hybridization of the target molecule with the probe, avoiding all the additional procedures currently required of manipulation, separation, washing, mixing and reading [3,4,5].

Biosensors are usually based on solid surface with probes immobilized as in the case of graphene-2D layered [6], magnetic particles [7], particles [8,9], carbon nanomaterials [10] microarrays [11]. The immobilization of probes and their presentation is a fundamental step in the process of biosensor design and realization and often the cause of shortcomings in the oligonucleotide probes recognition [12]. In particular, on solid-surface the electrostatic repulsion between probe-probe [13], together with the steric hindrance between probe-target duplex [14] and the non-specific oligonucleotides adsorption, affect the hybridization efficiency. As regards the hybridization rate, the design of probe, its length and GC content play a crucial role [15,16]. Furthermore, based on the immobilization methods biosensors suffer from inhomogeneous signal distribution and low conjugation reproducibility [17].

The ideal biosensor would combine the advantages of assays performed in solution with those obtained on solid surfaces. To achieve this goal, it is necessary to optimize the probe design and density, but above all, to fine tune the environment in which probes are immobilized. To tune these parameters three-dimensional (3D) functionalized materials represent the ideal choice. The 3D substrates offer higher capacity of immobilization compared to 2D surface. For similar probe concentrations conjugated, the crowding effects decrease in 3D substrates and more target/probe complexes are formed, increasing the sensitivity and reducing the background [18]. Additionally, tailoring the probe design and the linkage chemistry, probes are homogenously distributed into the whole volume and their density is finely tuned [19].

Furthermore, compared with 2D surfaces, the 3D-structures show larger available surface, achieving a faster probe diffusion with consequently more efficient recognition. Indeed, 3D systems allow to reach equilibrium in a reasonable time, conversely traditional planar microarrays show diffusional limitations due to poor mixing.

Moreover, since the environment of the 3D network influences the biosensor sensitivity and specificity, it must be as much as possible close to the ideal solution. For this purpose, the nature of the materials has a pivotal role, affecting both the diffusion of the molecules and the probe/target hybridization [20].

Polyethylene glycol (PEG) hydrogels are suitable materials due to their chemical flexibility, antifouling properties, biocompatibility [21], high water content and tuneable network structure. The tuneable network confers size exclusion ability as the polymer mesh size can be finely tuned to specifically allow diffusion and reaction of target molecules of different size, improving the assay sensitivity [22]. Moreover, it has been proved that a 3D functionalized polymer network offers not only enhanced sensitivity, but also better specificity, since probes spaced further apart hybridize less with mismatch sequences [16,23].

Among the various approaches for hydrogels synthesis, the microfluidic technique represents the most promising method for the production and functionalization of microparticles. Droplet microfluidics, in particular, reduce the costs, synthesis time and reagent consumption compared to the conventional techniques, achieving a large production of monodisperse microparticles per hour (about 105 particles) [24].

Based on these considerations, in this study we have designed 3D one step functionalized hydrogel microparticles by microfluidic for microRNA (miRNA or miR) detection. MiRNAs [24] are short non-coding RNA sequences, widely recognized as biomarkers in several diseases [25]. We have designed a specific double-strand probe for miR-143-3p detection. miR143-3p is a biomarker with high clinical value, it has proven to be important for diagnosis and prognosis of various diseases. For instance, in different cancer types (breast, osteosarcoma and colon cancer) demonstrated its potential in the follow up in combination with other miRs or as single biomarker [26,27,28]. The same biomarker was found circulating in serum of amyotrophic lateral sclerosis (ALS) patients to be important in early diagnosis [29]. It has been moreover associated with metabolic syndrome conditions [30] and has been demonstrated how in HIV (Human Immunodeficiency Virus) and HCV (Hepatitis C Virus) infected individuals at different stages of disease progression miR143-3p could provide a new target for developing novel therapies [31] and a potential biomarker for viral hepatitis B and C [32].

The detection is based on hydrogel fluorescence turn-off following a double-strand displacement mechanism and is driven by the free energy of displacement gained following the target hybridization.

Herein, optimizations of the 3D PEG hydrogels microparticles structural parameters are performed together with diffusion study by molecular probes, to understand the relevant parameters for the subsequent assay. The results described in this study showed that 3D-hydrogels are specific for miR-143-3p detection and reached a limit of detection (LOD) of 30 pM. The 3D-PEG-hydrogels microparticles can be comprised into mix and read bioassays as they proved the ability to detect miRNA directly in human serum, just mixing microparticles with the sample and reading the fluorescence turn-off.

## 2. Materials and Methods

### 2.1. Fabrication of Engineered Microparticles

**Figure 1:**
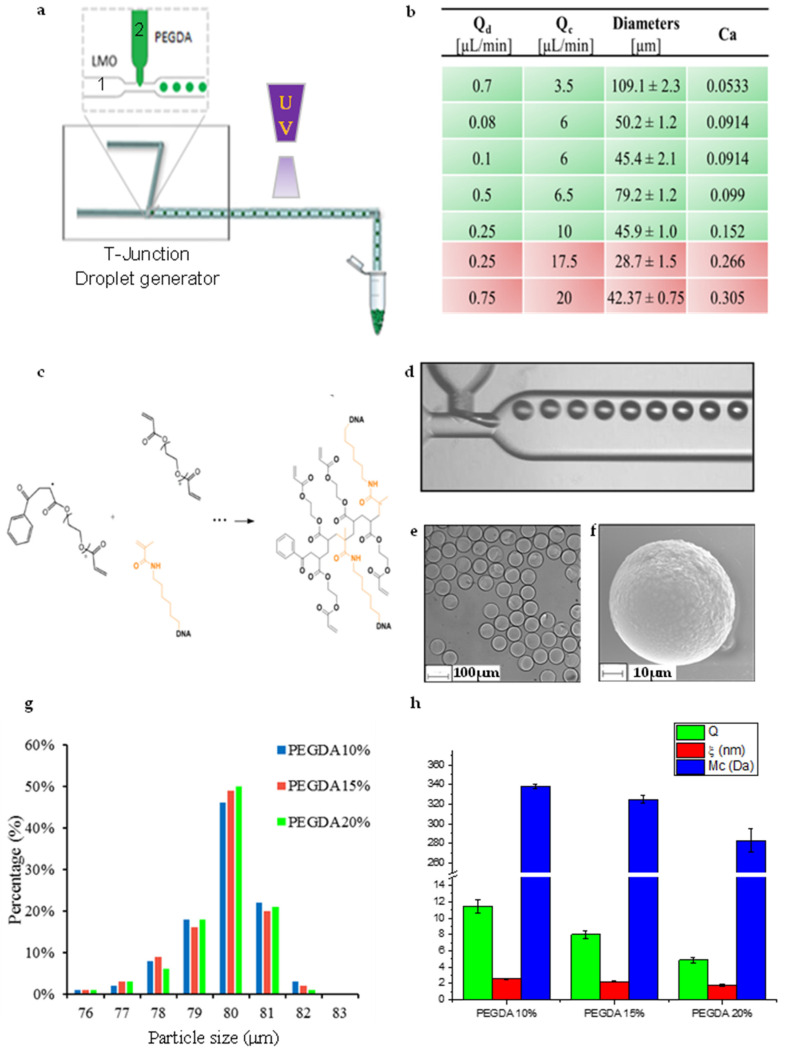
(**a**) Schematic representation of microfluidic synthesis set-up for microparticles production; (**b**) Table of PEGDA pre-polymer flow rate (Qd), LMO flow rate (Qc), diameters of water in oil emulsion and capillary number (Ca); (**c**) UV free radical photopolymerization between PEGDA and methacrylate oligonucleotide; (**d**) Droplet generation by microfluidic T-junction device; (**e**) Optical image of monodisperse microparticle; (**f**) SEM image of microparticle; (**g**) Size distribution of functionalized microparticles (PEGDA 10–15–20% *w*/*v*); (**h**) Swelling parameters (Q, ξ, Mc) for different polymer concentrations.

The synthesis of microparticles was performed using a glass microfluidic device from Dolomite (Figure 1). The chip consists of two inputs for the continuous and disperse phase, a narrow orifice where the two opposite channels converge, and an output. The dimensions of the device are 22.5 × 15.0 × 4 mm (length × width × thickness) with wide channels cross-section of 100 × 300 μm (depth × width) and channels cross-section at junction of 100 × 105 μm (depth × width) (Figure 1a,d). Microparticles were synthesized using light mineral oil (LMO, Sigma-Aldrich, Milano, Italy) containing non-ionic surfactant Span 80 (5% *v*/*v*, Sigma-Aldrich) as a continuous phase and a water solution of poly(ethylene glycol) diacrylate (PEGDA, MW 700 Da, Sigma-Aldrich) (10–15–20% *w*/*v*) with the photoinitiator 2-Hydroxy-2-methylpropiophenone (0.1% *v*/*v* with respect to the total volume, Darocur 1173 Ciba) as dispersed phase. Methacrylate oligonucleotide (Metabion GmbH, Steinkirchen, Germany) was added in the dispersed phase to reach final concentration of 1 μM into the water solution. Reaction occurs by a UV free radical photopolymerization between PEGDA and oligonucleotide, properly modified with methacrylamide moieties (Figure 1c). Droplet emulsions were obtained adding in the first channel water solution and continuous phase in the other. Solutions were injected using high-precision syringe pumps (neMesys-low pressure) to ensure stable flow and reproducibility. This system was mounted on an inverted microscope (IX 71 Olympus, Tokyo, Japan). The droplets formation was monitored using an objective with 5× magnification and 0.12 numerical aperture and recorded with a CCD camera ImperxIGV-B0620M that allows recording up to 259 frames per second. Once emulsion was formed, it was cross-linked outside the chip using an UV lamp at 365 nm wavelength at 850 mW power lamp for about 2 min obtaining a complete polymerization. Thereafter, microparticles were collected in an eppendorf and washed several times with different solvents (diethyl ether, ethanol and milliQ water-tween solution (0.05% *v*/*v*)) in order to remove all the residual oil and surfactant. After washing, microgels were stored at room temperature in buffer solutions until further use. Our buffer was obtained mixing 1× PBS (MP Biomedicals, Irvine, CA, USA), NaCl (Sigma-Aldrich) 200 mM and tween-20 (Sigma-Aldrich) at 0.05% *v*/*v* in milliQ water.

### 2.2. Microparticles Characterization

The production of monodisperse and stable emulsion is deeply affected by several parameters such as flow rates (i.e., the pre-polymer flow rate (Qd), and LMO flow rate (Qc)), the viscosity of the fluid and the capillary number (Ca) (Figure 1b). In order to achieve high controlled microparticles, all these physical parameters were properly optimized, and best conditions were investigated. Droplets’ diameters were calculated in flow during the droplet production before and after polymerization showing similar results (Figure 1g).

**Figure 2:**
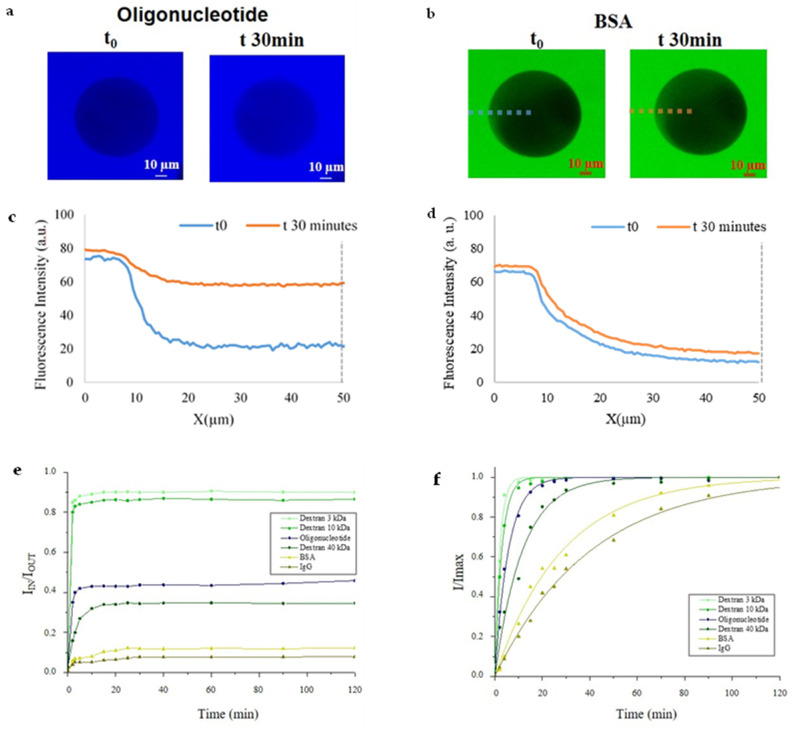
CLSM images at different time points (0 min and 30 min) for (**a**) Oligonucleotide and (**b**) BSA. Plot profile of fluorescence intensity of Oligonucleotide (**c**,**d**) BSA Protein. Diffusion in microparticles: (**e**) Time lapse of fluorescence intensity (I_IN_/I_OUT_) of several probes. (**f**) Time lapse of fluorescence intensity (I/Imax) of several probes. All values reported show a standard deviation of 10%.

For swelling characterization, approximately 100 microparticles diluted in 20 μL were loaded onto μ-slide 18 well-flat (IBIDI, Martinsried, Germany). Images in both swollen and dry conditions were collected with CLSM microscope (CLSM Leica SP5 with an Objective HC PL FLUOROTAR 20 × 0.5 DRY and a scan speed of 8000 Hz). Then, images were analyzed by ImageJ software to obtain diameter and calculate volume of microparticles in both conditions in order to apply equilibrium swelling theory [33,34] (Supplementary Materials). Morphological characterization was done collecting images by SEM and CLSM. The SEM measurements were performed on a FE-SEM Ultra Plus (Zeiss, Oberkochen, Germany‎) microscope at 20 kV. For sample preparation, the microparticles solution was fixed on a microscope slide, air-dried and then sputtered with a 10 nm thin gold layer. Diffusion characterization was carried out following fluorescence intensity changes by CLSM (Figure 2). In particular, 10 μL of microparticles solution, containing approximately 100 microparticles, were loaded onto μ-slide 18 well-flat with 10 μL of probe solution: (a) 2 µM dextrans 3, 10, 40 kDa (Thermo Fisher Scientific, Waltham, MA, USA); (b) 2 µM Target miRNA-143-3p; (c), (d) 2 µM Albumin-fluorescein isothiocyanate conjugate (BSA) and Anti-Human IgG (whole molecule)-FITC antibody produced in goat (IgG) (Sigma-Aldrich). Samples were illuminated at CLSM Leica SP5 using appropriate wavelength (Helium-neon λ_ex_ 633, Helium-neon λ_ex_ 543, Argon λ_ex_ 488 nm) and fluorescence images of microparticles were collected.

### 2.3. Probe Design and Hydrogel-Beads Based Assay Set-Up

Oligonucleotide probes were designed for selective and specific miRNA detection according to the double-strand (ds) displacement assay, based on fluorescence depletion in presence of target (Table 1). Alignment, folding and thermodynamic studies were carried on by the means of specific tools (BLAST, UNAfold, IDT (Integrated DNA Technologies Inc., Coralville, IA, USA) OligoAnalyzer 3.1. Web.” (2014)), in order to design oligonucleotide probes with enhanced target recognition capability, stability and appropriate difference in free energy of displacement. All the probes and synthetic targets (DNA/RNA) were purchased from Metabion with HPLC purification and re-suspended in water (Sigma Aldrich Molecular biology grade). The DNA probe was designed based on the miRNA-143-3p sequence (21 nt), and its length was properly optimized to achieve higher stability, specificity and Δ*G* of displacement in presence of the target. For this purpose, a short DNA tail (t-DNA, 12 nt) was modified with a methacrylamide spacer at the 3′ end, for covalent bound within polymer network while a longer fluorescent capture DNA sequence (F-DNA, 21 nt), complementary to the specific target, was labelled with the dye ATTO 647N at 5′ end. (Table 1) When the F-DNA and t-DNA partially hybridize into the hydrogel, the particle becomes fluorescent. In presence of the target, the F-DNA and the target hybridize and diffuse out of the hydrogels so that microparticles fluorescence decreases. Fluorescence intensity was measured by CLSM with λ_ex_ 633 nm λ_em_ 650–700 nm.

**Figure 3:**
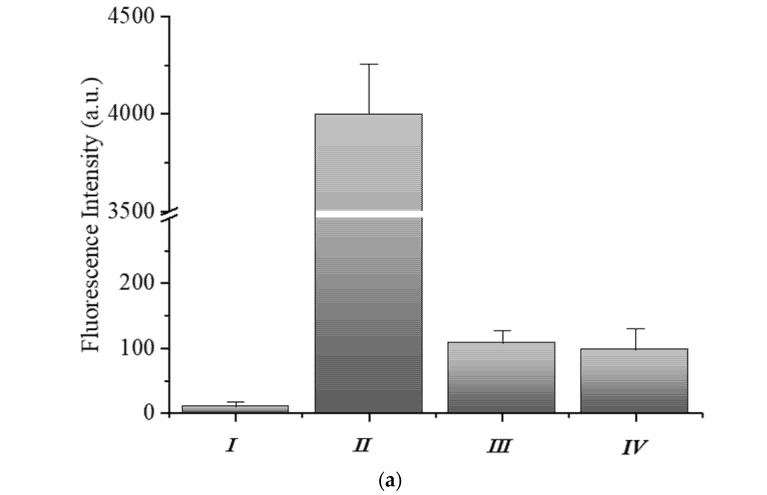

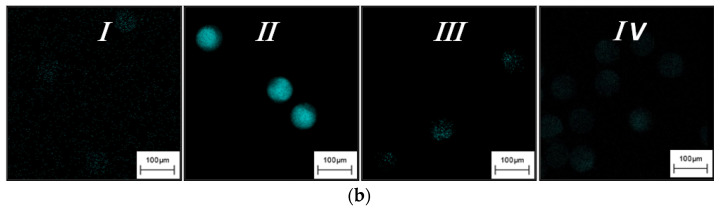
(**a**) Fluorescence intensity measured during the hydrogel-based assay setup and (**b**) the corresponding CLSM images. In particular, the figure shows the fundamental steps involved in the target detection: (I) after the synthesis of functionalized hydrogel; (II) when the fluorescent DNA strand was added; (III) in presence of the target in hybridization buffer and (IV) after target hybridization in human serum.

**Figure 4:**
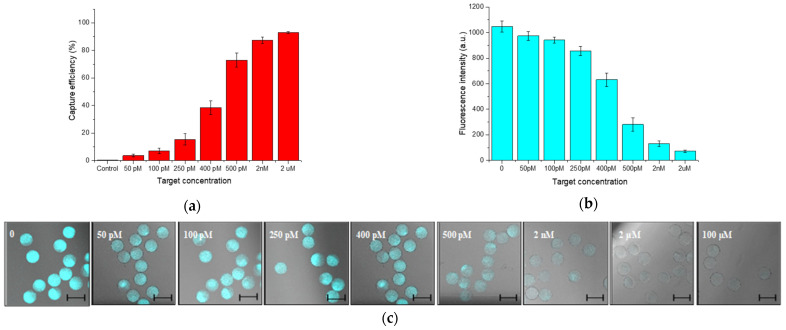
(**a**) MiR143-3p capture efficiency by Hydrogel beads assay and (**b**) corresponding fluorescence intensity turn-off (**c**) Images collected by CLSM for the target concentration analyzed (Scale bars: 100 μm).

The hydrogel-beads based miRNA assay consists of two steps (Figure 3): (I) fluorescent capture strand (F-DNA) hybridization and (II) Target hybridization step. All experimental steps were performed at room temperature in both hybridization buffer (1× PBS, NaCl 200 mM and tween-20 at 0.05% *v*/*v*) and human serum (Lonza). Regarding the first step (I) 100 μL of microparticles-Tail solution (~25 × 10^3^ particles and ~6 pmol of Tail), were put in contact with F-DNA 10× (~60 pmol) in 1.5 mL DNA-low Bind tube (epperdorf). Solution was kept under stirring until use for three days and then washed several times with hybridization buffer. Finally, for the CLSM experiment, 1 μL of this solution was diluted in 30 μL of buffer, added in μ-Slide VI 0.4 (IBIDI, Martinsried, Germany) and therefore the fluorescence intensity was evaluated. Concerning the second step (II), several samples containing 2 μL of microparticles-Tail-F solution (~330 particles and ~60 fmol of Tail), were loaded in DNA-low bind tubes with different Target concentrations (from 100 μM to 50 pM in buffer solution) in order to define the limit of detection (LOD) (Figure 4). The particle performances were also carried out in human serum solution adding 200 μL of serum to 300 μL of hybridization buffer where Target 2 µM was spiked in. The solution was kept under stirring and washed several times with hybridization buffer before fluorescence analysis. For CLSM, 1 μL of this solution was diluted in 30 μL of hybridization buffer as described above.

### 2.4. Assay Specificity

**Figure 5:**
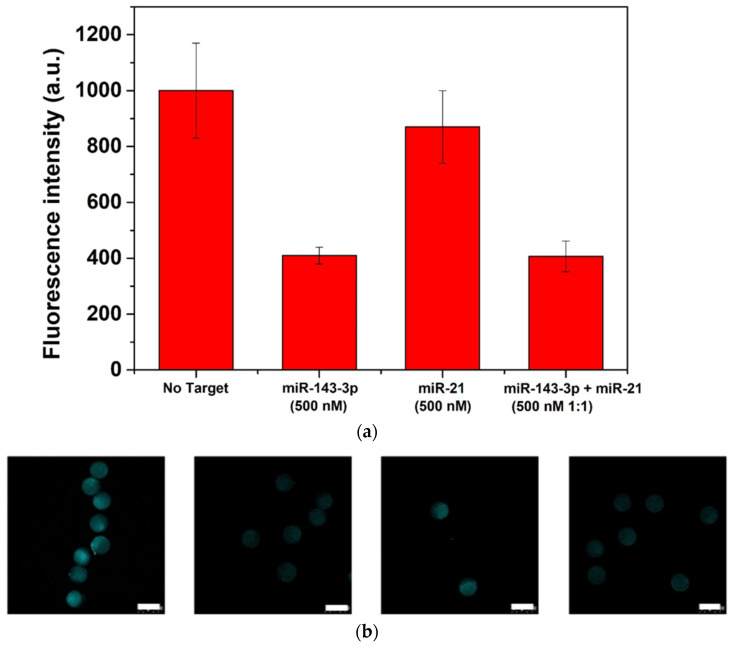
Assay specificity of 3D-hydrogel microparticles: recovery of fluorescence (**a**) and corresponding confocal images (**b**) (scalebar corresponds to 100 μm).

The 3D-PEG-hydrogel microparticles were tested mixing 2 μL of microparticles-Tail-F solution (~330 particles and ~60 fmol of Tail) with 500 nM of non-matching sequence miR-21 or 500 nM of miR-21/miR-143-3p 1:1 ratio (Figure 5). The solution was incubated at room temperature overnight, then microparticles were washed three times with the hybridization buffer and analyzed by confocal microscopy as already reported.

## 3. Results and Discussion

### 3.1. Microparticles: Synthesis and Characterization

The optimization of physical parameters is a critical point for the synthesis of monodisperse microparticles. Among these, flow rates, viscosity of the fluid, capillary number (Ca) influence the droplet generation [35,36].

The synthesis of hydrogels microparticles was performed using a T-junction microfluidic device. The LMO was injected as continuous phase, while water solution of PEGDA, methacrylate oligonucleotide and photoinitiator was injected as dispersed phase (Figure 1a). Hence, we focused on the control of these parameters and, in particular, on the effect of capillary number (Ca) on the emulsion stability. This parameter deeply influences the droplet generation. As in fact, this formation is driven by a balance between viscous forces and surface tension acting across the interface between two immiscible fluids [35]. The Table 1 reported in Figure 1b show different flow-rate regimes with respective diameters and Ca values. Unstable emulsions are generated when Qc values were higher than 10 μL/min. Otherwise, for Qc between 6–10 μL/min, Ca was in a limited range 0.05~0.20 and the polymer phase broken into stable emulsions. These results are consistent with previous studies [36,37]. Indeed in these works the stability and monodisperse emulsions can be produced in T-junction device in Ca range of 10^−3^–10^−1^.

Microfluidic device allows to control over a wide range of sizes. Stable emulsions were produced with different sizes in the range of 46–110 μm. Droplet diameters calculated in flow during the droplet production before and after polymerization show similar results. A precise control of particle size and monodispersity are fundamental for several applications and, in particular, for a correct beads-based assay. Just outside the chip, the UV free radical photopolymerization occurs. The process involves three basic steps, briefly summarized in Figure 1c. Firstly, in the initiation reaction step, photoinitiator free radical was formed under UV light. Then, during the propagation step, the free radical comes in contact with the end of a PEGDA molecule and reacts with the carbon–carbon double bond in the acrylate functional group. This step produces a second free radical species, which can go on to react with more PEGDA polymers and/or methacrylate oligonucleotide species, propagating the crosslink until termination process occurs. The correct formation of network composed by PEGDA and methacrylate oligonucleotide was properly tested by fluorescence analysis by CLSM. (Supplementary Materials—Figure S1).

Microparticles were constantly monitored during the production step, images were recorded and their size was measured just after emulsion formation using Droplet monitor software (Dolomite Microfluidics, Royston, UK) (Figure 1d). Data analysis shows the average droplet size and their distribution, the spacing between droplet and droplet rate. For all particles (PEGDA 10–15–20% *w*/*v*) specific flow rates were chosen: 0.5 μL/min for dispersed phase and 6.5 μL/min for continuous phase obtaining a droplet rate of ~40 particles/sec. Once polymerized, particles were collected in a vial, washed and characterized by optical and electronic microscope. Images so obtained (Figure 1e,f) confirmed that microparticles were monodisperse and spherical, with a narrow diameter range of about 80 μm (Figure 1g). A complete swelling characterization was carried out on several samples obtained using three polymer concentrations, in order to better characterize a different network structure. In particular, swelling parameters (v2, s, Q, Mc, ξ) were calculated (Supplementary Materials—Table S1) and reported in Figure 1h. As expected, changing the PEGDA concentration, the equilibrium-swelling ratio decreased from 11.42–4.87 with PEGDA increasing. The molecular weight between crosslinks decreased from 338–283 Da as the crosslink density increased. With these recipes, mesh size in the range of 2.55–1.77 nm was achieved. All these results are due to the deep dependence between physical parameters and synthesis recipe. In fact, as in accordance with several studies done by Peppas and other research groups [20,33,38], as the amount of bifunctional monomers in the hydrogel increases, the degree of crosslinking increases and, thereby, decreases both the average molecular weight between crosslinks, the equilibrium-swelling ratio and mesh size. These results clearly demonstrate that we are able to fine tune the network just by regulating polymer concentration.

### 3.2. Diffusion Studies

Probe diffusion is strongly dependent on the size, shape and flexibility of the probe. Therefore, for our studies, we tested several probes with different molecular weight, hydrodynamic radius and flexibility (Supplementary Materials—Table S2). Furthermore, probes were properly chosen to simulate the behavior and diffusion of biological molecules. We tested dextrans with different molecular weight (3, 10 and 40 kDa). The smallest probe is dextran 3 kDa, chosen due to its hydrodynamic radius, which is comparable with our target radius. On the other hand, high molecular weight dextran (40 kDa) reproduces molecules that we want to exclude from our network. Then we tested our target, miRNA 143-3p to compare its diffusion behavior with small dextran diffusion. Finally, we tested blood proteins (albumin) and antibody (IgG) which show hydrodynamic radii similar with high molecular weight dextran but more rigid structure, representing, in the same way, molecules that we want to exclude.

Dextrans are slightly branched polysaccharides, usually chosen as spherical probes in diffusion experiments, even though they are flexible, porous macromolecules. Instead, proteins such as BSA and IgG, were used as hard spheres [39,40].

For our diffusion studies, we focused on PEGDA 15% microparticles functionalized with 1 μM DNA-Tail. These, in fact, can combine both network structure and stability required for the detection. We excluded PEGDA 20%, because of its narrow mesh size (as demonstrated by swelling characterization) that resulted to be not suitable for the diffusion of our probes. Otherwise, PEGDA 10% showed a high swelling ratio (Q) that entailed an evident mechanical instability during our washing steps. Diffusion percentage was evaluated following the fluorescence intensity changes by CLSM images. In Figure 2d are reported plot profiles of fluorescence intensities for both oligonucleotide (Figure 2a–c) and proteins (Figure 2b–d). Intensities values of all probes tested were reported in graph as ratio between the fluorescence intensity inside the microparticles (I_IN_) and fluorescence in the surrounding gel (I_OUT_) (Figure 2e–f). As reported in Figure 2e we noticed that ~85% of both 3 and 10 kDa dextrans were able to spread into microparticles. A smaller percentage, ~40%, of 40 kDa and oligonucleotide are capable to diffuse into the hydrogel. Finally, only the 10% of proteins (BSA and IgG) were detected in the network. According to Cheng et al. [41], dextrans show a flexible structure, caused by a partial branching that allow them to diffuse in narrow mesh size. Comparing the diffusion behavior of 3 kDa dextran with methacrylate oligonucleotide, our studies show that despite similar hydrodynamic radii, dextran diffusion is facilitated by its flexible structure which allows it to diffuse quickly into the particles. Analogously, a very different behavior is shown by diffusion of 40 kDa dextran and rigid globular proteins such as BSA. These results show, as expected, that our particles work as a molecular filter allowing the diffusion in 30 min of oligonucleotide and, instead, leaving out large molecules such as proteins.

Moreover, intensities values were reported in graph as ratio between the fluorescence intensity inside (I_IN_) and maximum fluorescence reached in the microparticles (I_max_). In Figure 2f is compared the diffusion behavior of all probes inside the microparticles, reporting I/I_max_ versus time fitting following Equation (1).

(1) $$\frac{I}{I_{\max}}=1-e^{-\frac{t}{\tau }}$$

where *τ* is the extrapolated value that represents time required to achieve 67% of maximum intensity. In such a way, we were able to determine *τ* for each curve, Dextran 3 kDa 2.1 ± 0.1 min, Dextran 10 kDa 2.9 ± 0.1 min, Oligonucleotide 5.7 ± 0.3 min, Dextran 40 kDa 11.6 ± 0.7 min, BSA 28.4 ± 1.2 and IgG 40.4 ± 2.0 min. Consequently, smaller probes required about 2–3 min to accomplish 67% of maximum value. Oligonucleotide needed ~6 min while both proteins needed longer times, approximately 30–40 min. Because *τ* is inversely proportional to the diffusion coefficient, we have calculated that the oligonucleotide diffusion coefficient (D) into the hydrogel microparticle is 26.6 × 10^−8^ cm^2^/s. This value is comparable to that observed for free oligonucleotide in solution (115 × 10^−8^ cm^2^/s for oligonucleotide of 23 bases) [38] and confirms that the 3D-PEG- hydrogel environment is comparable to free solution. Furthermore, analyses carried out in this study have proved the molecular size exclusion capability of our microparticles. To sum up, our results showed that it is possible to use our engineered microparticles as molecular filter allowing the target to diffuse and excluding most large molecules, such as proteins.

### 3.3. Human-miR-143-3p Detection in PBS and Human Serum

In this study, the target recognition is based on a double-strand displacement assay, quantified by the microparticle fluorescence turn-off. The double strand probe consisted of a short naked methacrylate DNA-Tail (T-DNA, 12 nt), covalently bound into the polymer networks during the polymerization step, and a longer partially complementary fluorescent DNA sequence (F-DNA, 21 nt) added in a second step. In presence of the totally complementary target the F-DNA strand hybridized with it, moving in the solution, with a consequent fluorescence decrease into the hydrogel.

Figure 3 shows each step of hydrogel beads-based assay and the relative analysis of fluorescence. Reported below are referred to as an excess of Target (2 μM). The fluorescence of the T-DNA hydrogel is measured to evaluate the background signal that resulted very low, as shown in Figure 3a,b(I). Then F-DNA (10-fold excess) is added to the T-DNA particles solution, incubating until equilibrium was reached. The significant increase in microgels fluorescence observed in the Figure 3a,b(II) confirmed that hybridization occurred.

When the target is spiked into the solution, it quickly diffuses into the 3D hydrogel network) and driven by the favorable Δ*G* of displacement (Table 1), it hybridizes with the complementary F-DNA strand diffusing out of the hydrogel. This event causes a decrease of the hydrogel fluorescence as reported in Figure 3a,b(III). Additionally, we tested the assay performance in complex fluids, such as human serum. Therefore, even in this case, hydrogels confirmed good performances achieving about 93% of target detection (Figure 3a,b(IV)).

In order to define the assay working range, the limit of detection (LOD) and the limit of quantification (LOQ), several target concentrations were tested ranging from 10^−6^–10^−12^ M. Results, reported in Figure 4a–c, show an efficient working range comprises between 2 µM to 50 pM. When 2 μM of target is used, the capture efficiency is 93.1 ± 0.76%. Reducing the target concentrations down to 500 pM, we reach 73 ± 5.09% of detection, which decreases until 3.7 ± 1.09% at 50 pM. The LOD and LOQ were calculated as reported in Supplementary Materials (Figure S3) and their values were respectively 30 and 91 pM.

To achieve such fluorescence depletion, our probes were appropriately designed in terms of alignment, folding and thermodynamics. From the alignment studies we have predicted that not-specific complementarity is avoided. Based on the analysis of the probe folding prediction, we opportunely tailored the length of the probe in order to (i) corroborate the hybridization with the complementary strand and (ii) avoid self-secondary structures that could decrease their hybridization ratio (Supplementary Materials—Figure S2). Moreover, the thermodynamic parameters of our probes were investigated, in particular, the free energy of displacement Δ*G_disp_* calculated as:

(2) $$\Delta G_{disp}=\Delta G_{hybFT}-\Delta G_{hybFt}$$

where Δ*G_hybFT_* and Δ*G_hybFt_* are respectively the free energy of hybridization of the duplex formed by the F-DNA/Target and F-DNA/Tail-DNA strands. Such value was optimized in order to have favorable Δ*G_disp_* about −10 kcal/mol, enhancing the target displacement (Table 1).

The proposed assay, based on engineered hydrogel microparticles, has several advantages compared to conventional techniques. Indeed, 3D-PEG-hydrogels microparticles are synthesized and functionalized in microfluidic higher concentrations reducing time and cost of production. Furthermore, they might be directly mixed into the human sample without any previous oligonucleotide purification or amplification, and the target is easily quantified by reading the fluorescence depletion associated with the displacement event.

There are different techniques developed so far to detect and quantify with amplification free approached miRNAs. One very recent example provides a proof of concept detection of model miRs (miRNA-16b and miRNA-25) by an AC electrokinetic capacitive sensor [42]. A fast detection with a low LOD (about 1 femtomolar (fM)) was reached with high specificity. Such approach proved very sensitive though the sample might be diluted in low salt solutions as the charged species can affect the background. Instead, it is often important to have materials that are low fouling and can be used on the sample with less manipulation and pretreatment. As a result, our approach uses materials that are a good candidate to allow for direct detection in complex systems (like serum/plasma) as previously demonstrated [43,44].

### 3.4. Assay Specificity

Additional studies have been carried out on the 3D-hydrogels to investigate the assay specificity. We have selected the miR-21 as non-specific miR, since the miR-143-3p and the miR-21 have almost 50% of sequence similarity (Table 1) and the miR-21 is circulating in many central nervous system diseases [43,44]. For the purpose, 3D-hydrogel microparticles were put in contact with 500 nM of miR-21, then images were collected and analyzed (Figure 5a,b). From the fluorescence intensity measurements, it resulted that in presence of high concentration of non-specific microRNA (Table 1) a slight fluorescence intensity decrease is measured when compared with the control. Nevertheless, 500nM of miR-143-3p achieves a much stronger response than the blank. Furthermore, when combined solutions of miR-143-3p/miR21 500 nM (1:1) are tested the displacement performances measured are similar to those observed in absence of the non-specific target. This suggests that the designed probe has good selectivity toward miR-143-3p and the hydrogel microparticles are capable of detecting the target even in presence of highly similar oligonucleotide solutions.

## 4. Conclusions

In conclusion, here we report aG hydrogel bead-based assay for the direct detection of miRNA. Microfluidic allows to generate stable and monodisperse microparticles enabling high control of size, composition, chemistry and swelling parameters. Our results pointed out that microparticles synthesized with PEGDA 15% and T-DNA 1 μM best matched both stability and network structure for an efficient hybridization. Diffusion studies demonstrated the filter effect and the size exclusion of larger molecules with the possibility to select among the appropriate size of target molecule. An accurate design of probe enabled the 3D recognition of miRNA 143-3p bringing to its quantification in complex fluids, such as human serum. A displacement percentage of ~90% confirms the possibility to adapt hydrogel microparticles with tuneable network combining both molecular filter (allowing the target to diffuse and excluding most of big molecules) and detection capability. Our innovative assay represents a highly promising approach for the development of a non-invasive screening test. Moreover, the versatility of our assay proved the possibility to use these engineered hydrogels for the early detection and the monitoring of several diseases.

## Figures and Tables

**Table 1:** Sequence, length and thermodynamic parameters of the DNA probes and RNA targets. TF*_hyb_* represents the free energy gained from the partially complementary T-DNA/F-DNA duplex; FTarget*_hyb_* is the free energy gained from the fully complementary Target/F-DNA duplex; Δ*G_displacement_* is the free energy gained after T-DNA/F-DNA de-hybridization and Target/F-DNA hybridization.

Probe	Sequence (5′–3′)	Length (nt)	Δ*G* (Kcal/mol)
T-DNA	GCA CTG TAG CTC	12	TF*_hyb_* = −12.76
F-DNA	TGA GAT GAA GCA CTG TAG CTC	21	FTarget*_hyb_* = −22.62
Target	GAG CUA CAG UGC UUC AUC UCA	21	Δ*G_displacement_* = −9.86
Non-specific sequence	UAG CUU AUC AGA CUG AUG UUG A	22	

## Supplementary Materials

The following are available online at https://www.mdpi.com/article/10.3390/s21227671/s1, Figure S1: CLSM images of Microparticles; Table S1: Swelling parameters; Table S2: Diffusion probes parameters; Figure S2: Folding simulation of the Tail and Quencher strands; Figure S3: LOD analysis.
