# Supplementary material for: Hydrogel Microparticles for Fluorescence Detection of miRNA in Mix-Read Bioassay
Source: Sensors (Basel). 2021 Nov 18;21(22):7671. doi: 10.3390/s21227671 (PMC8624599; doi:10.3390/s21227671)
Supplement: Supplementary file 1 [file sensors-21-07671-s001.zip › sensors-1437152-supplementary.pdf]

sensors

MDPI

*Supplementary Material*

# Hydrogel Microparticles for Fluorescence Detection of miRNA in Mix-Read Bioassay

**Alessia Mazzarotta [1,†], Tania Mariastella Caputo [1,†], Edmondo Battista [2,*], Paolo Antonio Netti [1,2,3] and Filippo Causa [1,2,3]**

[1] Center for Advanced Biomaterials for Healthcare, Istituto Italiano di Tecnologia, L.go Barsanti e Matteucci, 53, 80125 Naples, Italy
[2] Interdisciplinary Research Centre on Biomaterials (CRIB), Università degli Studi di Napoli "Federico II", P.le Tecchio 80, 80125 Naples, Italy
[3] Dipartimento di Ingegneria Chimica del Materiali e della Produzione Industriale, Università degli Studi di Napoli "Federico II", P.le Tecchio 80, 80125 Naples, Italy
* Correspondence: edmondo.battista@unina.it
† All the authors equally contributed to this work.

**Citation:** Mazzarotta, A.; Caputo, T.M.; Battista, E.; Netti, P.A. and Causa, F. Hydrogel Microparticles for Fluorescence Detection of miRNA in Mix-Read Bioassay. *Sensors* **2021**, *21*, 7671. https://doi.org/10.3390/s21227671

Academic Editor: Ian Sandall

Received: 13 October 2021
Accepted: 16 November 2021
Published: 18 November 2021

**Publisher's Note:** MDPI stays neutral with regard to jurisdictional claims in published maps and institutional affiliations.

**Copyright:** © 2021 by the authors. Licensee MDPI, Basel, Switzerland. This article is an open access article distributed under the terms and conditions of the Creative Commons Attribution (CC BY) license (http://creativecommons.org/licenses/by/4.0/).

## Reagents and Chemicals

Poly(ethylene glycol) diacrylate (PEGDA-700 Da), Light mineral oil (LMO), Sorbitan monooleate (SPAN 80), Polyethylene glycol sorbitan monolaurate (TWEEN 20), Ethanol and Diethyl ether, Albumin-fluorescein isothiocyanate conjugate (BSA), Anti-Human IgG (whole molecule)-FITC antibody produced in goat (IgG) were purchased from Sigma-Aldrich (St. Gallen, CH) and used as received. Crosslinking reagent 2-Hydroxy-2-methylpropiophenone (DAROCUR 1173) was supplied from Ciba. Phosphate Buffered Saline Tablets were purchased from MP Biomedicals. Texas Red labelled dextrans with different molecular weight (3 kDa, 10 kDa and 40 kDa) were supplied by Thermo Fisher Scientific. DNA and RNA oligonucleotides were provided from Metabion with HPLC purification. Human serum was supplied by Lonza. μ-Slide were provided by Ibidi. Tube and special DNA-LoBind tubes were provided by Eppendorf.

## Microparticles: Synthesis and Characterization

The correct formation of network composed by PEGDA and methacrylate oligonucleotide was properly tested by fluorescence analysis by CLSM. In **Error! Reference source not found.** are reported PEGDA microparticles (15% w/v) used as control (CTR) and functionalized PEGDA–methacrylate microparticles. Both were properly analyzed by CLSM images at different time. Synthesized and washed microparticles were firstly examined at t0. Both of them show only a low background fluorescent signal. Then ($t_1$) a solution with F-DNA was added and, therefore, a high fluorescent signal in and outside the microparticles was recovered. Finally, microparticles were properly washed and re-analyzed. As expected, at t2, only functionalized microparticles show fluorescent signal due to the duplex formation between T-DNA and F-DNA confirming the correct formation of oligo-polymer network.

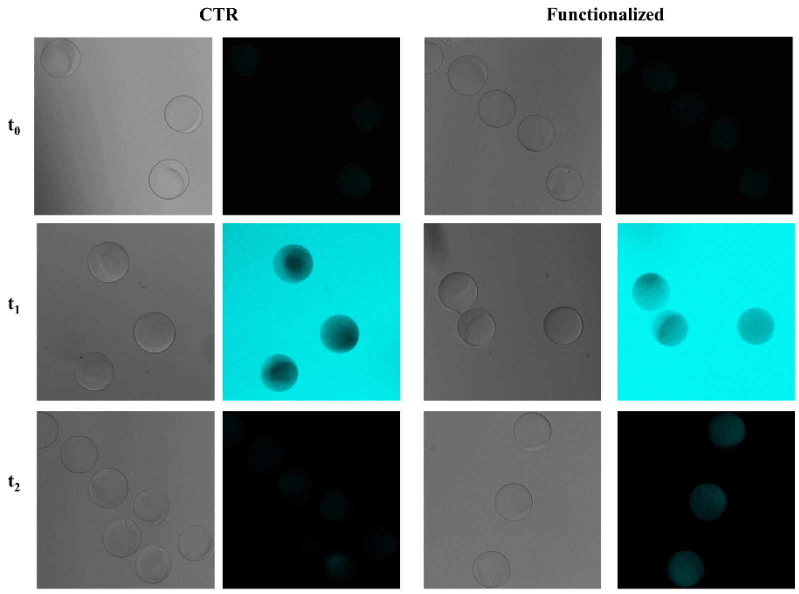


**Figure S1.** CLSM images of both control PEGDA microparticles (CTR) and functionalized ones at different times.

## Equilibrium Swelling Theory

The equilibrium swelling theory, also known as Flory-Rehner theory, allow analyzing the structure of hydrogels that do not contain ionic moieties. This equation allows calculating the molecular weight between crosslinks that represent a measure of the degree of crosslinking of the polymer. Obviously, due to the random nature of the polymerization process itself only average values of $\overline{M}_c$ can be calculated by Equation (S1).

$$\frac{1}{\overline{M}_c} = \frac{2}{\overline{M}_n} - \frac{\left(\frac{\bar{v}}{V_1}\right)\left[ln\left(1-v_{2,s}\right)+v_{2,s}+\chi_1 v_{2,s}^2\right]}{\left(v_{2,s}^{1/3}-\frac{v_{2,s}}{2}\right)} \quad \text{(S1)}$$

In this equation $\overline{M}_n$ is the molecular weight of the polymer chains prepared in the absence of a crosslinking agent, $v_{2,s}$ is the polymer fraction in the swollen state, $\chi_{1,2}$ is the polymer-solvent interaction parameter, $\bar{v}$ is the specific volume of the polymer, and $V_1$ is the molar volume of water.

In particular, polymer volume fraction in the swollen state characterizes how well the polymer absorbs water. It can be expressed as the ratio of the volume of dry polymer, $V_d$, to the volume of the swollen polymer gel, $V_s$, and is the reciprocal of the degree of swelling, Q.[101] The polymer fraction in the swollen state can easily be determined with equilibrium swelling using Equation (S2) above.

$$v_{2,s} = \frac{V_d}{V_s} \quad \text{(S2)}$$

To apply Equation (S1), several parameters are needed: interaction parameter PEG/water, $\chi$=0.426 (18); specific volume, v=0.91 ml/g; molar volume of water, V1= 18.1 ml/mol (18); uncrosslinked molecular weight of PEG, $M_n$ = 7000 Da.

Once $M_c$ was calculated, the mesh size ($\xi$) can be determined from the following equation [15]:

$$\xi = v_{2,s}^{-1/3}(\bar{r}_0^2)^{1/2} \quad \text{(S3)}$$

where $\bar{r}_0^2$ is the value of the end-to-end distance of PEG chains in the unperturbed state and it is calculated applying Equation (S4):

$$\langle r_0^2\rangle^{1/2} = C_n N l^2 \quad \text{(S4)}$$

where $C_n$ is the Flory characteristic ratio, l is the length of each link and N, the number of links in the chain, is given by Equation (S5):

$$N = \frac{2\,\bar{M}_c}{M_r} \quad \text{(S5)}$$

where $M_r$ is the molecular weight of the repeating unit of the polymer.

## Swelling Parameters

Table with all swelling parameters calculated applying Equilibrium Swelling theory for three different sample preparations ( PEGDA 10-15-20% w/v).

**Table S1.** Swelling parameters for different polymer concentrations.

| | PEGDA 10% | PEGDA 15% | PEGDA 20% |
|---|---|---|---|
| $V_{2,s}$ | 0.088 ± 0.007 | 0.125 ± 0.007 | 0.206 ± 0.014 |
| **Q** | 11.42 ± 0.87 | 7.99 ± 0.46 | 4.87 ± 0.33 |
| **Mc** (Da) | 338 ± 2 | 325 ± 4 | 283 ± 12 |
| ξ (nm) | 2.55 ± 0.10 | 2.23 ± 0.07 | 1.77 ± 0.09 |

## Diffusion Probes

**Table S2.** Diffusion probes parameters: Molecular weight (MW), Hydrodynamic radius (Rh) and structure.

| | MW (kDa) | Rh (nm) | Structure |
|---|---|---|---|
| **Dextran 3kDa** | 3 | 1.3 | Spherical, flexible, branched |
| **Dextran 10kDa** | 10 | 2.4 | Spherical, flexible, branched |
| **Dextran 40kDa** | 40 | 3.9 | Spherical, flexible, branched |
| **Methacrylate Oligonucleotide** | 6 | 1.6 | linear |
| **BSA** | 66 | 3.5 | Globular, rigid |
| **IgG** | 150 | 5.0 | Y-Shaped, rigid |

**Double Strand Probe**

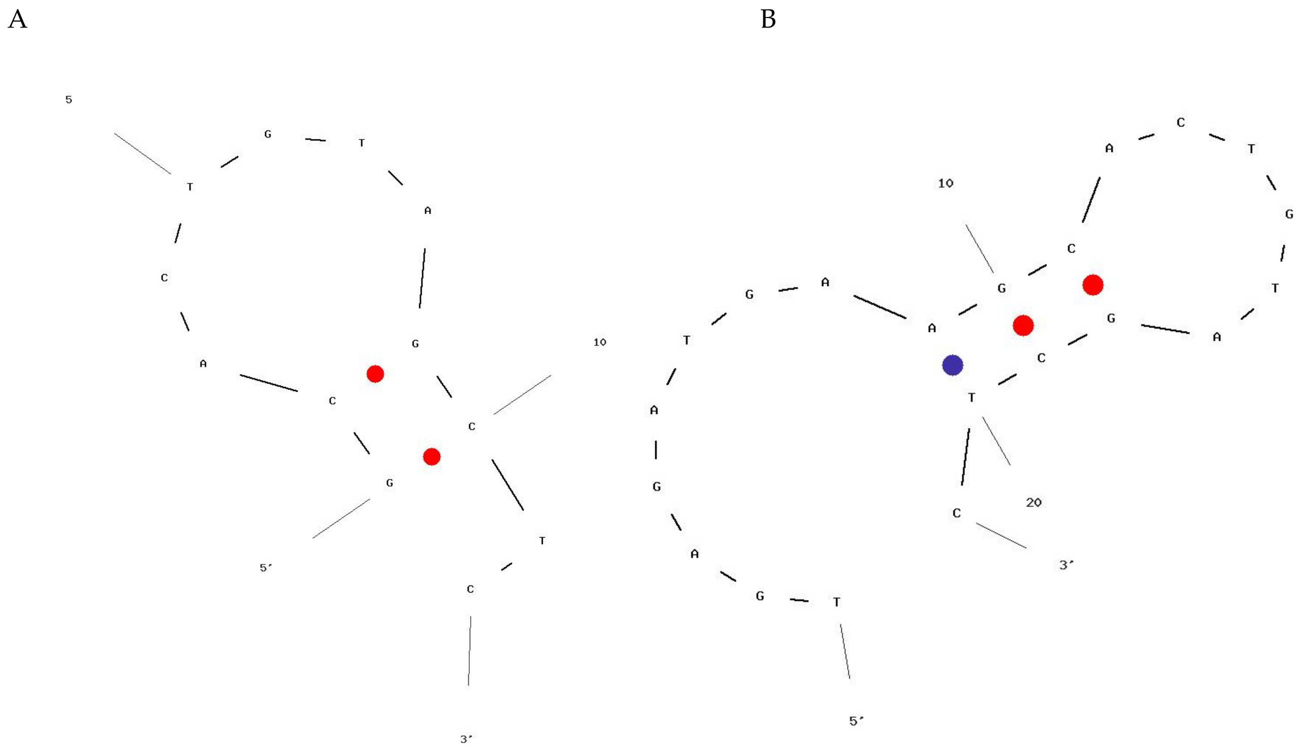


**Figure S2.** Folding simulation of the Tail (**A**) and Quencher (**B**) strands using UNAFold and setting 50 nM of oligonucleotide, 200 mM of NaCl and 25 °C as parameters.

**Limit of Detection Analysis**

All experiments were performed in triplicates and values are reported as mean ± standard deviation. Data are analyzed applying a non-linear regression and R2 is above 0.9 in all experiments.

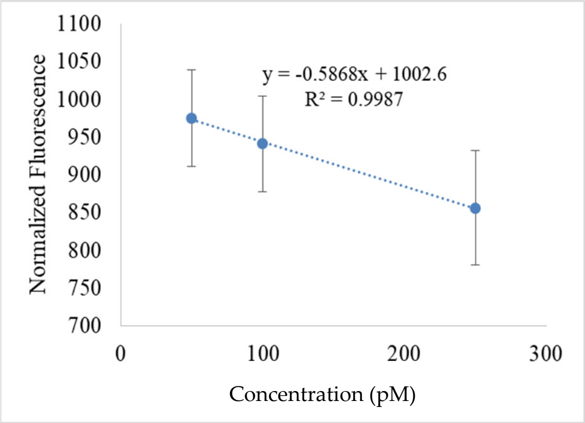


**Figure S3.** LOD analysis.
